# Activity impairment, work productivity, health-related quality-of-life, and costs associated with uncomplicated urinary tract infection among females in Germany

**DOI:** 10.1371/journal.pone.0313752

**Published:** 2025-01-07

**Authors:** Megan O’Brien, Alen Marijam, Fanny S. Mitrani-Gold, Laura Terry, Gavin Taylor-Stokes, Ashish V. Joshi

**Affiliations:** 1 Adelphi Real World, Macclesfield, United Kingdom; 2 GSK, Wavre, Belgium; 3 Department of Epidemiology, GSK, Collegeville, Pennsylvania, United States of America; Johns Hopkins Medicine, UNITED STATES OF AMERICA

## Abstract

Uncomplicated urinary tract infections (uUTIs) represent a sizable healthcare burden with a substantial negative impact on patients’ health-related quality-of-life (HRQoL). However, the HRQoL impact of uUTI from the patient perspective is under-represented in research. A cross-sectional online survey assessed activity impairment and work productivity, HRQoL, costs, and treatment satisfaction of female patients with uUTIs in the US; the current study applies this earlier methodology to Germany to provide a European perspective. We examined: activity impairment and work productivity using Activity Impairment Assessment (AIA), and Work Productivity and Activity Impairment (WPAI) questionnaires, respectively; HRQoL using a modified Short Form-36, and participants completed the treatment satisfaction questionnaire for medication. Participants (≥ 12 years) self-reported a uUTI treated with oral antibiotics in the last 60 days, had no evidence of complicated UTI, and were recruited via a consumer panel. In the patient (N = 200) survey, bladder pain (70%), dysuria (69%), and frequent urination (66%) were the most common uUTI symptoms reported. Activities frequently affected by uUTI were sexual activity (56%) and exercise (48%), resulting in an overall AIA score of 8.2 indicating that activities among patients were impaired ‘most of the time’ during their recent uUTI. Absenteeism was 29% (of a 40-hour work week); presenteeism, 34%; and overall work impairment, 41%. Indirect costs due to impact of uUTI were €7.62/hour/participant, representing a loss for the employer. The lowest HRQoL scores were for mental (44%) and physical components (43%); emotional and physical functioning scores were 71% and 70%, respectively. Mean direct costs of €20.10 for medical treatments and €11.30 for other treatment costs were reported. Overall, most participants were satisfied with the uUTI treatment (76% satisfaction score). Results demonstrate the patient-reported burden of uUTI for patients and employers, with activities and ability to work impacted, resulting in a notable indirect burden in Europe.

## Introduction

Uncomplicated urinary tract infections (uUTIs) are among the most common bacterial infections in women [1] and account for a substantial portion of outpatient antibiotic prescriptions [2–4]. Patients usually present with symptoms such as dysuria, urinary frequency, urinary urgency, and/or suprapubic pain [5]. Urinary tract infection (UTI) affects approximately 11% of the overall population, but increases to 20% in women aged ≥ 65 years of age [6]. The clinical significance of UTIs is well-documented, but these infections also represent an economic burden for healthcare systems and have a negative impact on patient quality-of-life (QoL) determinants, which has not been well studied [7]. The societal cost of UTI–including healthcare costs and time missed from work–is estimated to be over $5 billion per year in the US [8]. Despite the prevalence of uUTI and its associated issues, studies describing the impact of uUTI from the patient perspective are under-represented in uUTI research.

We conducted a survey to assess the impact of uUTI from a patient perspective in Germany. The objective of our study was to gain insights into the drivers of activity impairment, work productivity, health-related QoL (HRQoL), treatment satisfaction, and costs for patients with uUTI in Europe. Our methodology replicates a previous study from the US in which views of patients with uUTI were assessed [9]. Conducting similar studies in different geographical regions may deliver insights into region-specific differences in patients’ experience of uUTI, especially given that treatment guidelines and treatment options differ between the US and EU.

## Methods

### Participants

For the survey, female residents of Germany aged ≥ 12 years with a self-reported uUTI that had been treated with oral antibiotics in the last 60 days were recruited via a consumer panel provided by Dynata and RaPa. As characteristics and comorbidities associated with complicated UTI (cUTI) may have an additional impact on patients’ daily life separately from UTI, participants with suspected cUTI were excluded according to the following criteria: the presence of urologic abnormalities, ureteral abnormalities, undergone urological surgeries, interstitial cystitis, pyelonephritis, kidney stones, renal failure, congenital urological abnormalities, organ transplant, neurological disease, and immunosuppressive disorders (such as cancer or diabetes). Participants were also excluded if they were pregnant, if their self-reported uUTI had occurred during an inpatient hospitalization or a stay at a long-term care facility, if they received their initial uUTI-associated antibiotic therapy during an inpatient hospitalization, or if they were currently enrolled in a clinical trial.

### Survey

The survey was a cross-sectional, online survey conducted to assess the impact of uUTI on daily life and activities. Data were collected for a 7-month period from 26 February 2021 to 31 August 2021.

### Ethical approval and consent to participate

All participant information was deidentified prior to analysis and a centralized Institutional Review Board (Western Institutional Review Board-Copernicus Group [WCG]; Puyallup, WA, USA) reviewed the appropriateness of the methodological approach, and a waiver was provided. A statement of informed consent was provided during pre-screening and respondents were informed of their rights under general data protection regulation and other national, regional, and local laws pertaining to privacy and data protection. This study complied with all applicable laws regarding subject privacy and all methods were performed in accordance with the relevant guidelines and regulations (e.g., Declaration of Helsinki). Adults (≥ 18 years) provided written informed consent and for participants aged 12–17 years, the parent/guardian and the pediatric participant reviewed and consented to participation in the survey.

### Outcomes

The specific metrics evaluated in the survey were uUTI symptoms and their impact on everyday activities and productivity, work productivity and activity impairment, indirect costs, HRQoL, treatment satisfaction, and direct costs. Symptoms were self-reported by participants and symptom impacts were rated on a scale of 1–10 as follows: (1) not at all bothersome; (2–4) slightly bothersome; (5–7) bothersome; or (8–10) extremely bothersome.

Activity impairment was assessed using the Activity Impairment Assessment (AIA), and productivity loss was assessed using the Work Productivity and Activity Impairment (WPAI) questionnaire [10, 11]. The AIA is a validated, self-administered five-item questionnaire that assesses the amount of time that activities have been impacted by UTI symptoms as a score from 0–20 (higher scores indicate greater levels of disruption) [10]. The WPAI questionnaire yields four types of scores for employed participants: absenteeism (percentage of work time missed), presenteeism (percentage of impairment at work), overall work impairment (absenteeism plus presenteeism), and total activity impairment [11]. Study participants were asked to “think back to your recent UTI” when answering AIA- and WPAI-related questions. The standard AIA and WPAI questionnaires have recall periods of 24 hours and 7 days, respectively; in the present analysis, the standard recall period for both instruments was extended to 60 days. Absenteeism per week was estimated by participants, thinking back to their most recent uUTI. Indirect costs were calculated using WPAI scores and Federal Statistics Office of Germany hourly wage data from 2022 [12].

HRQoL was assessed using the Short Form-36 questionnaire version 2 (SF-36) to measure outcomes across eight key domains (emotional, physical functioning, social functioning, mental health, general health, physical, vitality, and bodily pain) and via two summary scores based on the aggregation of individual domain scores: the Mental Component Score (MCS) and Physical Component Score (PCS) [13]. SF-36 scores range from 0–100, with higher scores indicating better HRQoL; a score of 50 is considered a “normal” value [13].

Treatment satisfaction regarding the most recent oral antibiotic received for uUTI was assessed using the nine-item Treatment Satisfaction Questionnaire for Medication (TSQM-9) [14]. The TSQM-9 is scored from 0–100 (with higher scores indicating greater levels of satisfaction) in terms of three key domains (treatment effectiveness, convenience, and satisfaction) [14]. Direct costs included self-reported expenditures, such as over-the-counter treatment and costs associated with receiving treatment, including travel and childcare.

### Statistical analyses

Analyses were descriptive. The number of non-missing values, mean, standard deviation (SD), range, quartiles, and median were calculated for numerical variables. The number and percentage of participants in each category was calculated for categorical variables. All analyses were stratified by the presence or absence of recurrent uUTI (defined as ≥ 3 uUTI episodes in 12 months) [15] and the timing of uUTI (< 30 days ago versus 30–60 days ago). Sample size power calculations were not conducted due to the descriptive nature of analysis. The sample size was instead selected based on the intended precision of descriptive estimates, a reasonable recruitment length, and the associated cost of recruiting.

## Results

### Patient characteristics

A total of 200 patients completed the survey (Table 1). Most (n = 112; 56%) were aged 19–49 years, identified ethnically as White (n = 171; 86%), were employed (n = 138; 69%), lived in an urban community (n = 130; 65%), and lived with no children in the household (n = 146; 73%). Only 5% (n = 9) of participants were 18 years or younger. Most participants (n = 119; 60%) reported that they were diagnosed with their uUTI 30–60 days ago and the most common first antibiotic prescribed for the most recent uUTI was ciprofloxacin or fosfomycin (20% [n = 40] and 22% [n = 43] of participants, respectively). For 76% (n = 140/185) of participants, only one antibiotic was prescribed for their uUTI.

**Table 1:** Patient characteristics.

Parameter, n (%)	All patients (N = 200)
Age, years	
12–18	9 (5)
19–49	112 (56)
50–64	65 (33)
≥ 65	14 (7)
Ethnicity	
White	171 (86)
Mixed race	13 (7)
Other	4 (2)
Middle Eastern	4 (2)
Hispanic/Latin	5 (2)
Asian-other	2 (1)
Asian-Indian	1 (< 1)
Relationship status	
Married	85 (42)
Living with partner	44 (22)
Single, never married	41 (20)
Divorced	18 (9)
Separated	5 (2)
Widowed	5 (2)
Other*	2 (1)
Employment status	
Full-time employment	92 (46)
Part-time employment	38 (19)
Student	26 (13)
Homemaker	19 (10)
Retired	21 (10)
Self-employed	8 (4)
Unemployed	2 (1)
Education status	
Has attended school and/or professional/vocational training graduation	126 (63)
Attended university	61 (30)
Currently in school	9 (4)
Does not have a school or professional/vocational training graduation	4 (2)
Community setting	
Urban/City	130 (65)
Suburban	39 (20)
Rural	31 (16)
Number of children in the household	
0	146 (73)
1	24 (12)
2	24 (12)
3	4 (2)
≥ 4	2 (1)
Number of adults in the household	
1	48 (24)
2	122 (61)
3	20 (10)
≥ 4	12 (6)
Health insurance	
Public	179 (90)
Private	21 (10)

* Marital status classified as “other” were participants who declined to answer. Some percentages do not add up to 100 due to rounding.

### uUTI symptoms and their impact

**Fig 1:**
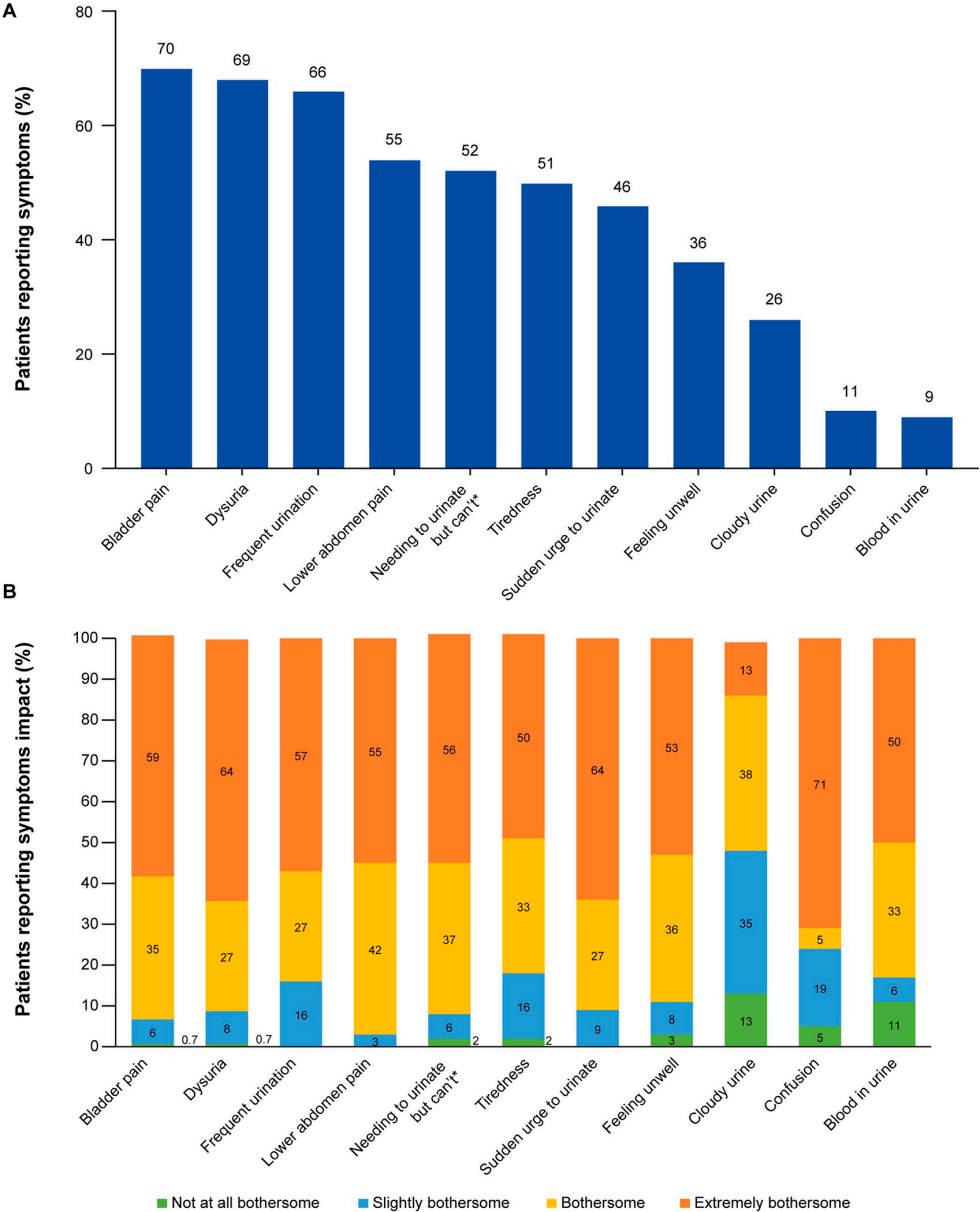
uUTI symptoms: (A) most reported symptoms and (B) the impact of uUTI symptoms. Note: symptoms impact = 0%, where no bar graph is shown. Participants rated the impact of each symptom on a scale of 1−10: (1), not at all bothersome; (2−4), slightly bothersome; (5−7), bothersome; (8−10), extremely bothersome. * Strong feeling of needing to urinate but can’t. Abbreviations: uUTI, uncomplicated urinary tract infection.

The uUTI symptoms reported by ≥ 50% of participants (N = 200) were bladder pain (n = 139; 70%), dysuria (n = 137; 69%), frequent urination (n = 131; 66%), lower abdomen pain (n = 109; 55%), needing to urinate but can’t (n = 104; 52%), and tiredness (n = 101; 51%; Fig 1A). Of the participants who experienced each uUTI symptom, confusion (n = 15 of 21 participants; 71%), dysuria (n = 45 of 137 participants; 64%), sudden urge to urinate (n = 58 of 91 participants; 64%), and bladder pain (n = 82 of 139 participants; 59%) were most reported as “extremely bothersome” (Fig 1B). Participants reported blood in urine (n = 2 of 18 participants; 11%) and cloudy urine (n = 7/52; 13%) as “not at all bothersome” (Fig 1B).

### Activity impairment

**Fig 2:**
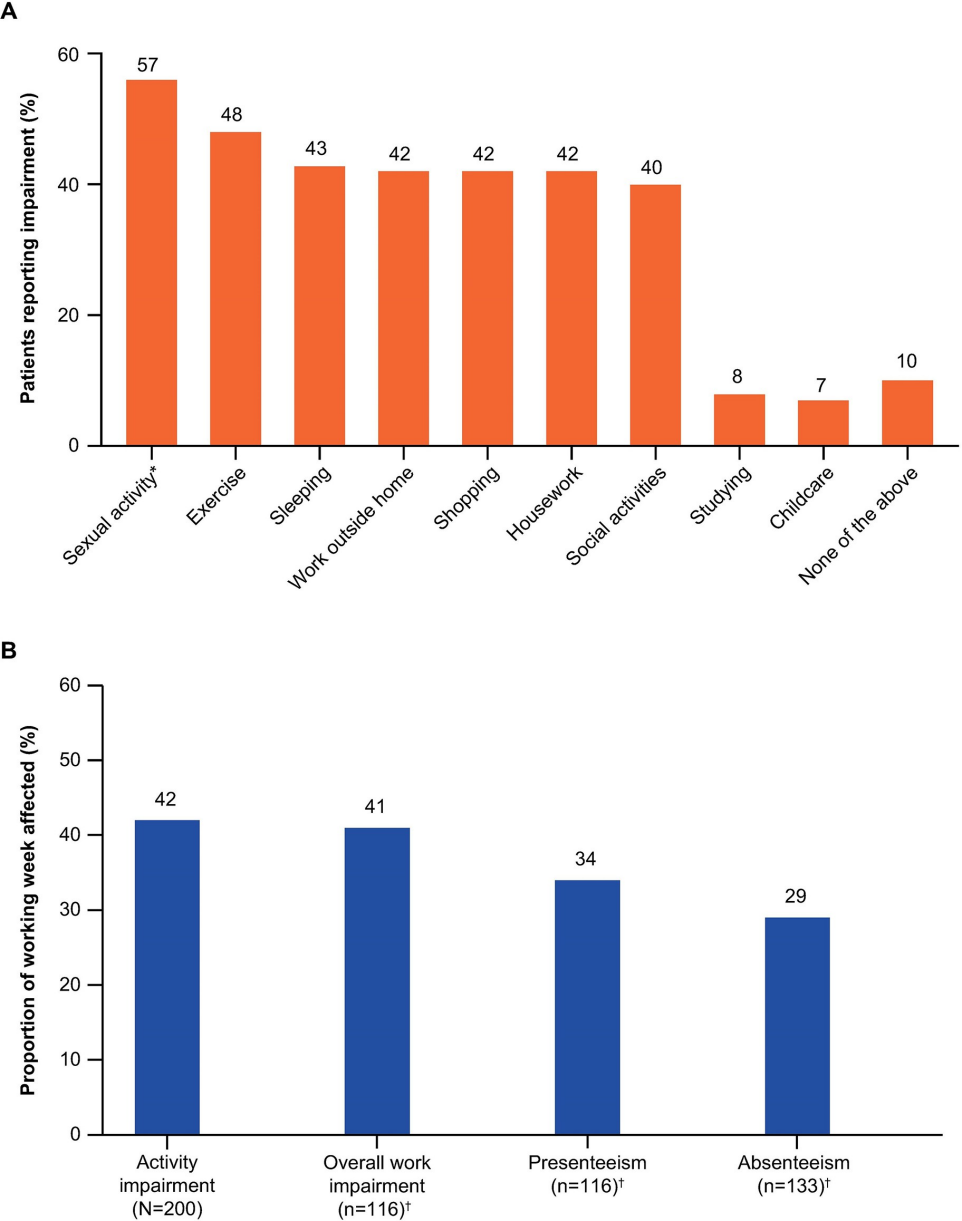
Activities and productivity: (A) everyday activities most commonly affected by uUTI, and (B) WPAI questionnaire results. * n = 197 (participants < 18 years were not asked about their sexual activity). ^†^ Note: participants who selected ‘Don’t know’ were not included in the analysis. Abbreviations: uUTI, uncomplicated urinary tract infection; WPAI, Work Productivity and Activity Impairment.

An AIA mean score of 8.2 (max 20) was observed across all participants, indicating that their activities were impaired “some of the time or more” by their most recent uUTI. The everyday activities reported by ≥ 40% of participants to be affected by uUTI were sexual activity (n = 112 of 197 participants; 57%), exercise (48% of all participants), sleeping (n = 85; 43%), work outside home (n = 83), shopping (n = 83), housework (n = 83) (all 42%), and social activities (n = 79; 40%). The least impacted activities were studying (n = 16; 8%) and childcare (n = 13 [6%]; Fig 2A). When participants rated the disruption to their daily activities due to their most recent uUTI on an increasing scale of 1–10, 21% (n = 41) gave a score of 8–10, suggesting a high level of impact.

### Productivity impairment

Of the 200 participants, 133 reported missing work; within a 7-day period, these 133 participants missed an average of 29% of a 40-hour work week (Fig 2B). While working, 116 participants reported that their most recent uUTI reduced their ability to work by 34% and overall work impairment was quantified at 41% for these participants; regular activity impairment for all participants was 42% (Fig 2B). The mean (SD) hours of work missed due to sick days and late starts/early finishes due to uUTI was 8.5 (12.3) hours for 134 participants and the mean (SD) hours worked was 24.9 (18.6) hours. When participants rated the disruption to their work activities due to their most recent uUTI on an increasing scale of 1–10, most responded with a three or less (1, 18%; 2, 18%; 3, 17%). The mean (SD) indirect cost of overall work impairment was €7.62 (€6.20) per hour per participant for 116 participants.

### HRQoL

**Fig 3:**
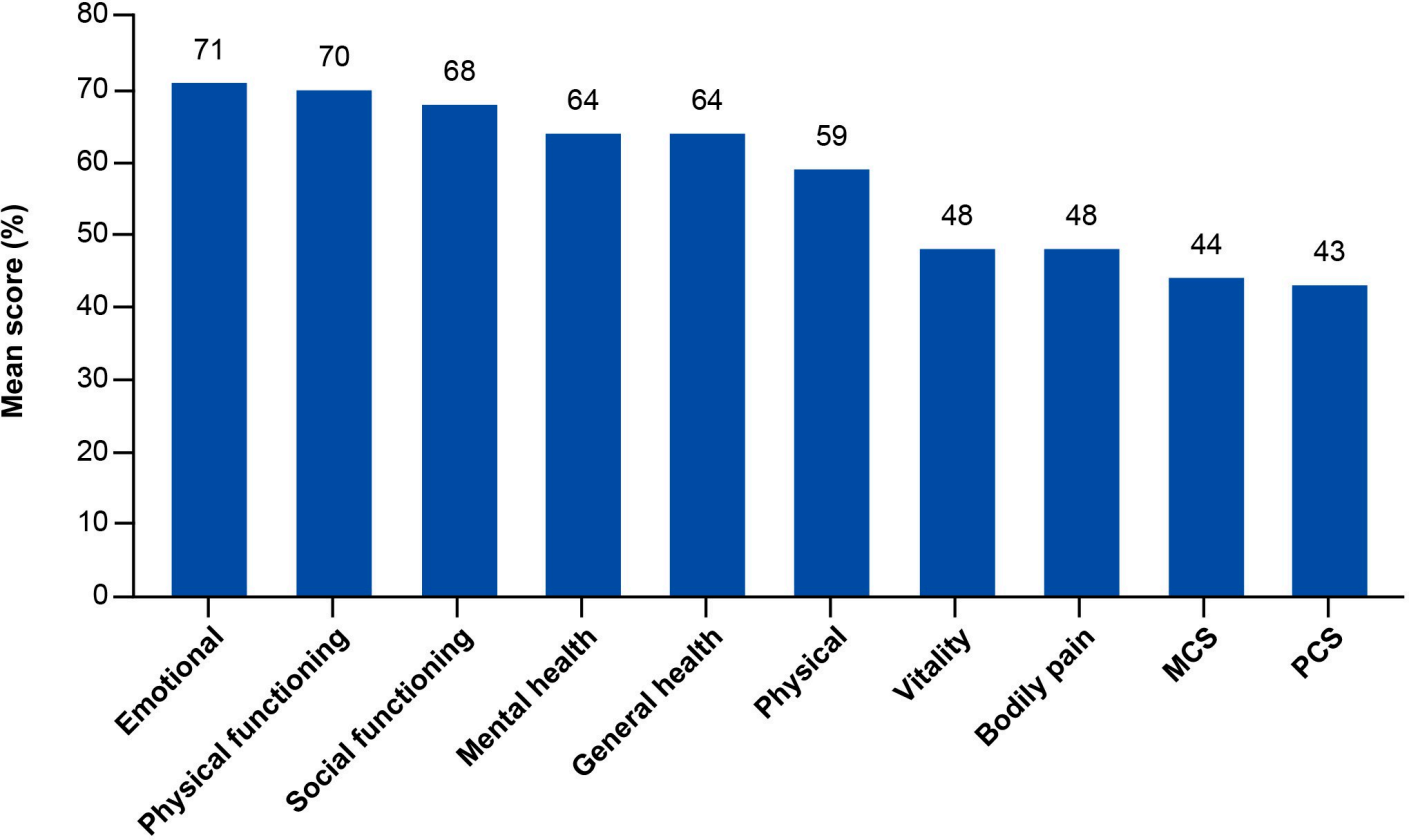
HRQoL per the SF-36. Abbreviations: HRQoL, health-related quality-of-life; MCS, Mental Component Score; PCS, Physical Component Score; SF-36, Short Form-36 questionnaire version 2.

Of the 200 participants, the highest scores in the SF-36 (0–100 = low–high HRQoL) were for emotional (71%), physical functioning (70%), social functioning (68%), mental health, and general health domains (both 64%; Fig 3). The lowest scores were recorded for the MCS and PCS (44% and 43%, respectively). When asked how their health now compared to their health 1 year ago, 81 participants (41%) reported that their health was “somewhat worse now than one year ago” and 15 (8%) reported that their health was “much worse now than one year ago”. Of the 200 participants, 64% reported that they were limited (a little or a lot) in conducting moderate activities such as moving a table, pushing a vacuum cleaner, bowling, or playing golf, and 84% reported that they were limited in conducting vigorous activities, such as running, lifting heavy objects, and participating in strenuous sports. Most participants reported moderate (n = 91; 46%) or severe (n = 49; 24%) pain during their most recent uUTI, but > 50% of participants stated that their pain did not interfere with their work (inside and outside of the home).

### Treatment satisfaction

**Fig 4:**
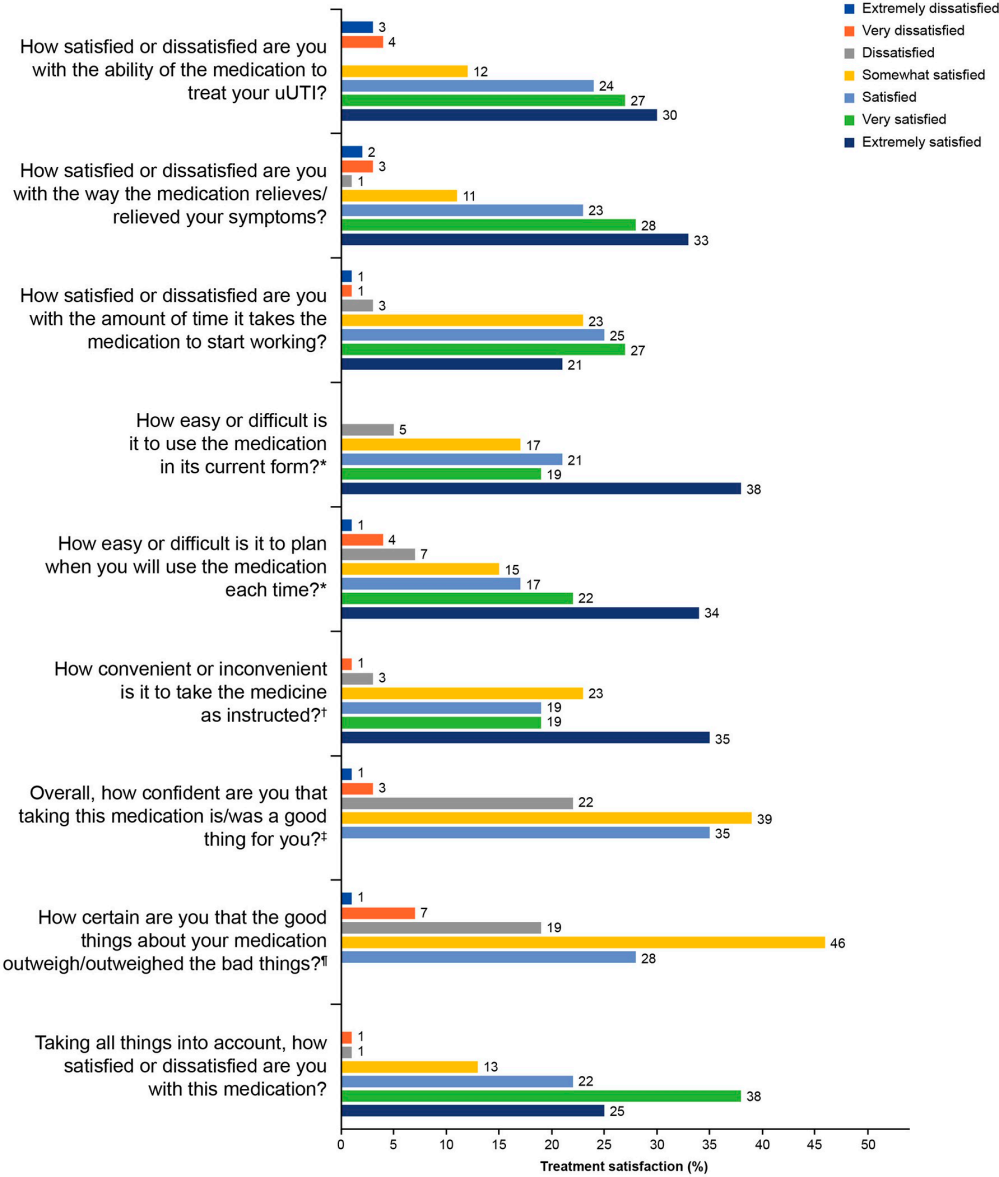
Treatment satisfaction per the TSQM-9 (n = 189). Note: Patients did not provide a response, where no bar graph is shown. * Response categories were: extremely difficult, very difficult, difficult, somewhat easy, easy, very easy, and extremely easy. ^†^ Response categories were: extremely inconvenient, very inconvenient, inconvenient, somewhat convenient, convenient, very convenient, and extremely convenient. ^‡^ Response categories were: not at all confident, a little confident, somewhat confident, very confident, and extremely confident. ^¶^ Response categories were: not at all certain, a little certain, somewhat certain, very certain, and extremely certain. Abbreviations: TSQM-9, 9-item Treatment Satisfaction Questionnaire for Medication; uUTI, uncomplicated urinary tract infection.

A total of 189 participants answered the TSQM-9 questions regarding treatment satisfaction. Treatment satisfaction was scored 76.5, convenience was scored 76.2, and effectiveness was scored 75.0. Many participants (n = 57; 30%) reported that they were “extremely satisfied” with the ability of their medication to treat their most recent uUTI and 71 participants (38%) reported that their medication was “extremely easy” to use (Fig 4). Taking all factors regarding their first-line medication into account, 48 participants (25%) reported that they were “extremely satisfied” with their medication; 72 (38%) were “very satisfied,” 41 (22%) were “somewhat satisfied,” one (< 1%) was “dissatisfied,” and two (1%) were “very dissatisfied.” The mean (SD) direct medical-related out-of-pocket costs for the treatment of participants’ most recent uUTI was €20.10 (€56.70) per participant (n = 129).

## Discussion

The patient survey was designed to assess the impact of uUTI on women aged ≥ 12 years in Germany. Participant demographics were broadly similar to the German female population, although study participants were more likely to have attended university and a lower proportion of the study participants were aged 65 years or older [16]. Participants reported considerable disruption to everyday activities and work productivity as a consequence of their most recent uUTI. The activities reported to be most disrupted in this study are aligned with those previously reported for patients with uUTI in the US (sexual activity, exercise, sleep, housework, and social activities), but the proportions of participants reporting each of these activities to be impacted was higher among US patients [9]. This increased disruption is reflected in the higher AIA score reported for US participants (11.1) versus the participants in this study (8.2) [9]. Disruption to sexual activities is a common complaint among female patients with UTI [17, 18]. One comment on the Cystitis and Overactive Bladder Foundation web forum read: “I am extremely depressed by the fact that we can no longer be intimate without weeks of pain afterwards” [19].

Regarding work productivity, levels of presenteeism, overall work impairment, and activity impairment were also higher in the US study by Thompson *et al*. (51%, 56%, and 55%, respectively) than in the current study (34%, 41%, and 42%, respectively) [9]. The indirect cost of the work impairment observed was €7.62 per hour per participant, which is almost one-third the average hourly wage in Germany (€22.65) and represents a significant loss from an employer perspective. A key difference between the published US study and the current study in Germany was that the productivity impact was reflected in greater presenteeism (51%) in the US, compared with greater absenteeism (29%) in Germany [9]. This suggests that US patients are more likely to go to work when suffering uUTI symptoms than those in Germany, who tend to stay home. This could be a function of cultural differences or benefit structure between the two countries. Further studies of the impact of uUTI on work productivity should be conducted to improve our understanding of the societal impact of UTI.

The mean MCS and PCS scores observed in this study (44 and 43, respectively) are similar to those reported in a recent US cross-sectional survey in women aged ≥ 18 years (47 and 40, respectively) [9]. Notably, the PCS observed in a matched general population in the US study was substantially higher than the PCS reported for German participants in the present study, which highlights the considerable impact of UTI on physical functioning [9]. This US study also demonstrated significant activity impairment alongside reduced work productivity and worse HRQoL for 375 participants with a recent uUTI versus a matched population from a 2020 survey [9]. Similarly, > 50% of 892 female respondents aged ≥ 16 years taking part in a 2014 computer-based household survey in England reported that their daily life had been affected “a fair amount” (37%) or “a great deal” (15%) when asked ‘To what extent, if at all, did your most recent UTI affect your daily life?’ [20].

The effects of uUTI on physical functioning were made clear in the current study as many participants reported difficulties in conducting moderate-to-vigorous activities and a worsening in their health in the past year. Despite the low MCS observed in this study, participants reported high scores for their emotional wellbeing (71%) and mental health (64%). These findings add to existing evidence for the negative impact of UTI on mental health among female patients, including a recent European survey that reported mental health scores up to 81% lower in patients with UTI versus comparators (physical health scores were up to 55% lower) [21].

The physical and mental impact of uUTI can be attributed in part to its most bothersome symptoms. A recent interview study with patients from Germany and the US identified feelings such as frustration, anxiety, and embarrassment associated with UTI symptoms and their impact on daily activities, relationships, and sleep [18]. The most commonly reported and most bothersome symptoms in the current study (pain, dysuria, and frequent urination) are typical of the UTI experience and are frequently reported by patients [22, 23]. The German/US patient interview study also highlighted emotions including frustration and anger with regards to treatment failure [18]. Participants in the current study were satisfied with their treatment, with > 80% reporting that they were satisfied, very satisfied, or extremely satisfied. The TSQM-9 scores obtained in this study (76.5 for treatment satisfaction, 76.2 for convenience, and 75.0 for effectiveness) are similar to those obtained in the US study by Thompson *et al*. (76.5, 82.8, and 70.8, respectively) [9].

Complicated UTI is associated with significant morbidity, carries a high risk of treatment failure, and a negative impact on patient wellbeing beyond that of uUTI [24]. However, survey-based quality-of-life studies of UTI frequently investigate UTI overall [17, 25]. Thus, a strength of this study is its focus on uUTI. A further strength is the breadth of outcomes evaluated. Additionally, this study complements the previous study by Thompson *et al*., which provided valuable insights into the factors affecting treatment decisions in real-world practice in the US, thus allowing both regional and more global insights [9]. According to our patient survey, ciprofloxacin and fosfomycin were the most frequently prescribed antibiotics as initial prescription and throughout uUTI. The use of fosfomycin as first-line treatment for uUTI is in accordance with the German treatment guideline, whereas, ciprofloxacin is recommended for the treatment of mild to moderate uncomplicated pyelonephritis and not for first line treatment of uUTI [26].

Limitations of the present study include the self-reported nature of the data, which has the possibility of recall bias and erroneous recording of recent antibiotic use. For example, participants may have received antibiotics for concurrent infections. Another limitation is that survey materials were developed for English and German speakers only. The German survey was translated from the English version, which, despite verification by an independent proof-reader, may have introduced errors or misunderstanding. In addition, this study extended the look-back period for validated WPAI and AIA from their standard durations of 7 days or 24 hours respectively, to up to 60 days, which may have affected the accuracy and validity of these outcomes.

## Conclusions

The impact of uUTI for female patients in Germany is multifaceted, including an impact on everyday activities, work productivity, and HRQoL. The most bothersome symptoms in this population–such as pain, dysuria, and frequent urination–are aligned with existing data from patients with uUTI in other countries. Our findings also demonstrate the employer burden of uUTI in Germany; this metric bridges the individual experience described through variables such as activity impairment, work productivity, and HRQoL with the wider societal consequences of uUTI.
